# Brief Report: Evaluating the Utility of Varied Technological Agents to Elicit Social Attention from Children with Autism Spectrum Disorders

**DOI:** 10.1007/s10803-018-3841-1

**Published:** 2018-12-03

**Authors:** Hirokazu Kumazaki, Zachary Warren, Amy Swanson, Yuichiro Yoshikawa, Yoshio Matsumoto, Yuko Yoshimura, Jiro Shimaya, Hiroshi Ishiguro, Nilanjan Sarkar, Joshua Wade, Masaru Mimura, Yoshio Minabe, Mitsuru Kikuchi

**Affiliations:** 1 Research Center for Child Mental Development, Kanazawa University, 13-1, Takaramachi, Kanazawa, Ishikawa 920-8640 Japan; 2 Departments of Pediatrics, Psychiatry and Special Education Vanderbilt Kennedy Center, Nashville, TN USA; 3 Treatment and Research Institute for Autism Spectrum Disorders, Vanderbilt Kennedy Center, Nashville, TN USA; 4 Department of Systems Innovation, Graduate School of Engineering Science, Osaka University, Toyonaka, Osaka Japan; 5 JST ERATO ISHIGURO Symbiotic Human-Robot Interaction, Osaka, Japan; 6 Service Robotics Research Group, Intelligent Systems Institute, National Institute of Advanced Industrial Science and Technology, Tsukuba, Ibaraki Japan; 7 Department of Mechanical Engineering, Vanderbilt University, Nashville, TN USA; 8 Department of Neuropsychiatry, Keio University School of Medicine, Tokyo, Japan

**Keywords:** Autism spectrum disorders, Technological agents, Robot, Android robot, Digital avatar

## Abstract

Technological agents could be effective tools to be used in interventions for enhancing social orienting for some young children with ASD. We examined response to social bids in preschool children with ASD and typical development (TD) at a very early age (i.e., around 3 years) using social prompts presented by technological agents of various forms and human comparisons. Children with ASD demonstrated less response overall to social bids compared to TD controls, across agents or human. They responded more often to a simple humanoid robot and the simple avatar compared to the human. These results support the potential utilization of specific robotic and technological agents for harnessing and potentially increasing motivation to socially-relevant behaviors in some young children with ASD.

## Introduction

Social communication deficits are core to autism spectrum disorders (ASD) (APA 2013). Described as social communication “building blocks,” (APA 2013) deficits in social orienting and joint attention skills are posited as central to the etiology and early neurodevelopmental sequelae of ASD (Dawson 2008; Poon et al. 2011). At a basic level, social orienting and joint attention refer to skills that involve sharing attention, particularly visual attention, with others (e.g. responding to a name call, coordinating gaze with social partner, responding to bids from others) (Mundy et al. 2010). These exchanges enable young children to socially coordinate their attention with other people to more effectively learn from their environments. There is growing empirical support suggesting that early intervention can systematically improve these early social communication skills and that such improvements partially mediate improvements in other critical developmental areas, including social and language outcomes (Poon et al. 2011).

Researchers have hypothesized that technological tools may be particularly promising as intervention mechanisms for some children, given that many young children with ASD (1) exhibit strengths in understanding the physical (object-focused) world and relative weaknesses in understanding the social (person-focused) world (Klin et al. 2009). (2) respond well to technologically cued feedback, and (3) show intrinsic interest in technology (Annaz et al. 2011; Diehl et al. 2012; Goodwin 2008; Klin et al. 2009; Pierno et al. 2008). However, to date most approaches have measured behavior in response to simple exposure to discrete and varied robots, toys, or screen based interactions (Duquette et al. 2007; Goodrich et al. 2011; Kim et al. 2012). As such, despite initial promising results there is very limited data about the type of robotic or technological stimulation that might be ideal for children with ASD.

Most children, including those with ASD, show an affinity to computer graphic displays (Ploog et al. 2012). Computer graphic displays can be harnessed to provide effective intervention for children with ASD (Grynszpan et al. 2013; Ploog et al. 2012). Studies have suggested several reasons for the special interest that many children with ASD demonstrate towards computerized learning. They have identified key advantages that computers provide with respect to difficulties in solving social problems and generating multiple solutions to the problems of ASD (Bernard-Opitz et al. 2001; Moore and Calvert 2000). Simultaneously, one consistent area of criticism and concern is the risk to increase dependence on computer-based activities that further impede social opportunities and skill development among children with ASD (Durkin 2010; Moore and Calvert 2000).

Social robots are autonomous physical agents, often with human-like features, that can interact socially with humans in a semi-naturalistic way and for prolonged periods of time (Dautenhahn 2007). Social robots can be good motivators for children with ASD (Lee and Obinata 2013; Lee et al. 2012a, b; Wainer et al. 2013, 2010, 2014; Yin and Tung 2013) because they show a clear attraction for technological systems. In some settings, robots have been shown to be a more effective stimulus for social behavior compared to humans (Pennisi et al. 2016) as well as effective therapeutic tools for language skills (Kim et al. 2012). With regard to imitation tasks, children with ASD performed better with a robot compared to children with TD (Cook et al. 2014; Pierno et al. 2008), while other studies have not shown this difference in joint attention tasks (Anzalone et al. 2014; Bekele et al. 2013). An advantage of using social robots, compared to computer based interactions, is that individuals can be exposed to a three-dimensional learning experience that more closely resembles situations in real life settings. For, example, in a previous study (Kumazaki et al. 2017b) that used a social robot for mock job interviews, it was found that the participants were exposed to a three-dimensional learning experience that simulated the challenging, and potentially anxiety-provoking situations, of job interviews in a controlled manner. Social robots with varying degrees of similarities to humans have potential to provide support for children with ASD in skill acquisition. However, to date, there has not been a comparison study examining types of technological agents best suited for this purpose.

With significant advances in technology and interest in robotics, the physical appearances of humanoid robots currently used in therapeutic settings is highly varied (Ricks and Colton 2010). Accordingly, robot developers and therapists are interested in identifying the optimal appearance for robots used in interventions, and have recently attempted to examine preference for robot appearance specifically in individuals with ASD. For example, “KASPAR” (Peca et al. 2014; Wainer et al. 2013, 2014) is a humanoid robot that has specific human-like features, but has been deliberately designed so that it is perceived as a robot. Wainer et al. (2014) evaluated whether individuals with ASD found “KASPAR” to be more interesting and entertaining than adult humans, and found that this was indeed the case.

When designing objects for use by children with ASD, researchers often subscribe to the notion that “simpler is better”, that is, children with ASD will gravitate toward simple, mechanical objects (Costa et al. 2014; Iacono et al. 2011; Ricks and Colton 2010; Robins et al. 2005, 2009; Wainer et al. 2013). On the other hand, our previous study suggested that preferences of robotic appearances may vary tremendously across children with ASD (Kumazaki et al. 2017c). The appearance of a robot is likely to be very important for designing tools that are efficacious in assisting children.

The aim of the present study is to offer a preliminary, yet systematic comparison between response rates of children with ASD and TD children to a range of technological agents as well as a human assistant. To do this, we examined how often preschool children with ASD and TD looked in response to social bids made by two types of humanoid robots, a digital avatar, and human. We utilized two varied robots for this protocol: human-like android robot with a face resembling an adult female and a humanoid robot which has simple face. We expected that children with ASD would (1) respond less than TD children across robot and avatar on the computer display, (2) respond at higher rates to both robots and avatar compared to the human, and (3) respond more often to a visually simple robots over the lifelike robot.

## Methods

### Participants

All children were recruited through existing university-based registries. Fourteen children with ASD (10 males; age [mean ± standard deviation]: 3.33 ± 0.93 years) and 23 children with TD (13 males; age: 3.19 ± 1.42 years) completed the study. One additional child with ASD was enrolled but unable to complete the study due to fear of the experimental set-up, and difficulty remaining in the experiment area. All children with ASD had received a clinical diagnosis of ASD based on Diagnostic and Statistical Manual Volume 5 criteria (APA 2013), and the diagnosis was confirmed using the Autism Diagnostic Observation Schedule, Second Edition (ADOS-2; Lord et al. 2012) by a licensed clinical psychologist. We evaluated language development of participants using the MacArthur-Bates Communicative Development Inventories (MCDI; Fenson et al. 2007). Parents of children in both groups also completed both the Social Communication Questionnaire (SCQ; Rutter et al. 2010) and the Social Responsiveness Scale, Second Edition (SRS-2; Constantino and Gruber 2002) to screen for clinically significant ASD symptoms in the TD group and as an index of current symptoms in the ASD group. Participant characteristics are described in Table 1.

**Table 1:** Participant characteristics

Mean (SD)	Age (years)	SRS-2	SCQ	CDI single word understanding raw
ASD mean (SD)	3.32 (0.93)	68.40 (7.12)	17.92 (3.88)	17.00 (29.66)
TD mean (SD)	3.19 (1.42)	41.36 (4.18)	4.00 (3.66)	62.27 (44.29)

*SD* Standard deviation, *SRS-2* social responsiveness scale—second edition, T-score, *SCQ* social communication questionnaire lifetime total score, *CDI* The MacArthur-Bates communicative developmental inventories

### Apparatus

Three technological agents were used in the study: two humanoid robots (one android robot and one simple humanoid robot) and a digital, screen-based avatar. Agents utilized in the study were novel to all participating children and none had any prior exposure to any of the technological agents.

**Fig. 1:**
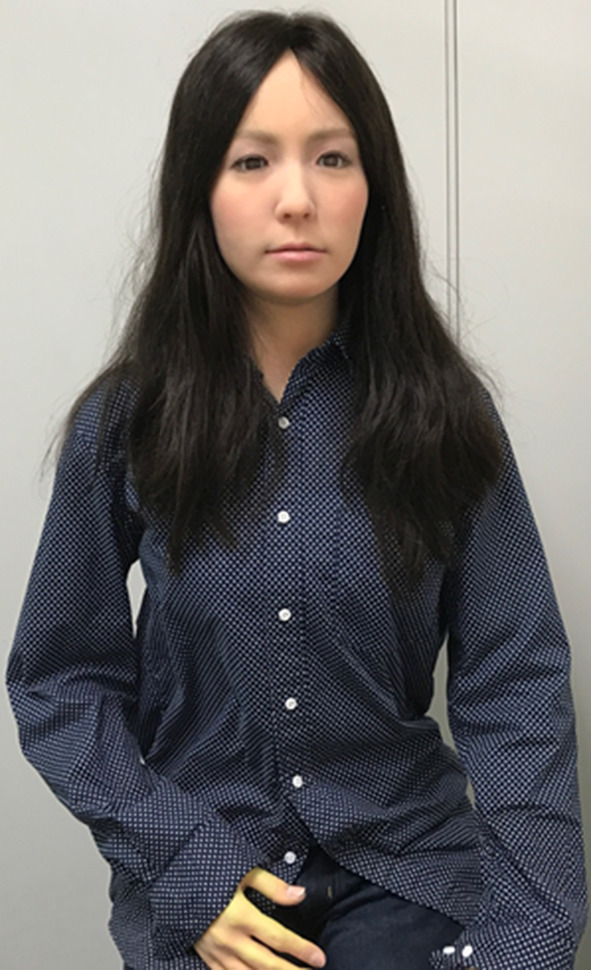
ACTROID-F (android robot)

The first humanoid robot is pictured in Fig. 1. “ACTROID-F” (Kokoro Co. Ltd.) is a female prototype of an android robot designed to replicate an appearance strongly similar to that of a real person (Kumazaki et al. 2017a, b, c, d). Its artificial body is designed with the proportions, facial features, hair color, texture and style as a human. It has clearly distinguishable eyes, with capacity to shift gaze, blink, and establish eye contact with humans in its environment. Its face can show a range of expressions, albeit in a less sophisticated way than a real human face.

**Fig. 2:**
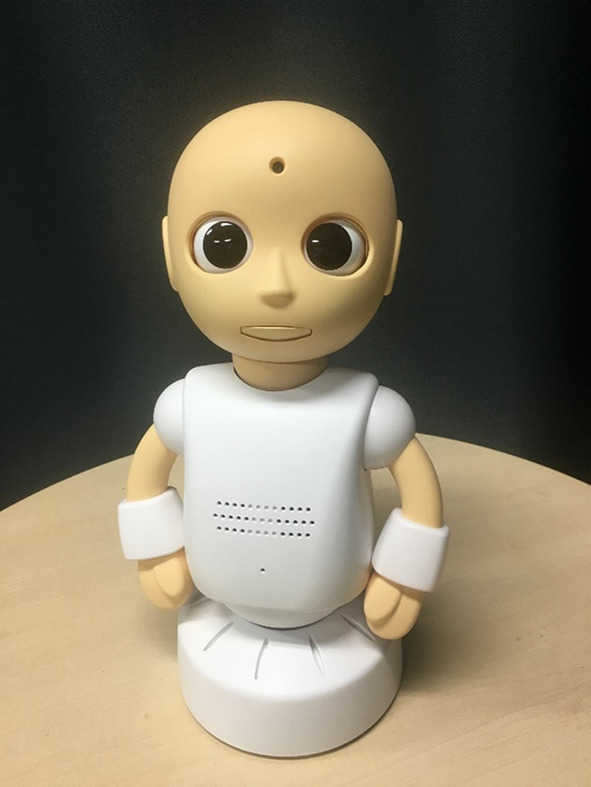
CommU (simple humanoid robot)

The second humanoid robot is pictured in Fig. 2. “CommU” (Vstone Co., Ltd.) is 304 mm in height (Shimaya et al. 2016). It has clearly distinguishable eyes, which are its most distinct and prominent feature. “CommU” is also capable of shifting gaze and blinking, and by way of smooth movement and positioning of its eyelids, can demonstrate a range of expressions, but in a less complex way than the android robot.

**Fig. 3:**
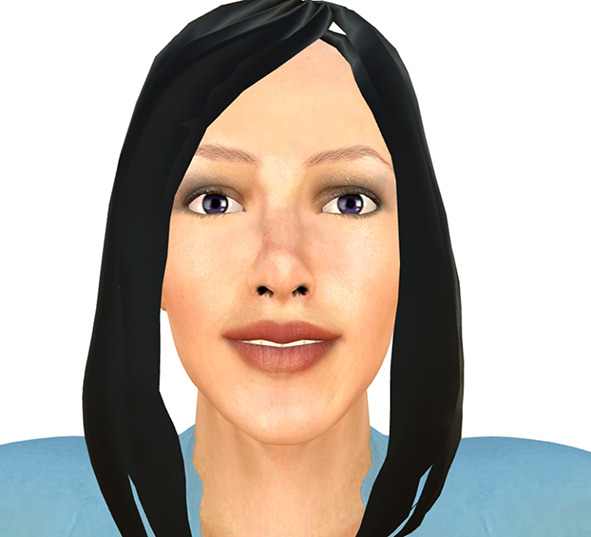
Digital avatar

The third technological agent utilized is a screen-based digital avatar (Fig. 3). Avatars are commonly used in virtual-reality based intervention systems. The avatar utilized for the present study was created using Unity, a cross-platform game engine developed by Unity Technologies (http://www.unity3d.com). The avatar designed was female in appearance, and displayed on a monitor mounted on the wall. The dimensions of the avatar’s face were: width = 18.38 cm; height = 31.40 cm.

For the human agent, we included several research assistants (22–26-year-old Caucasian females) to provide the social prompt.

### Procedure

**Fig. 4:**
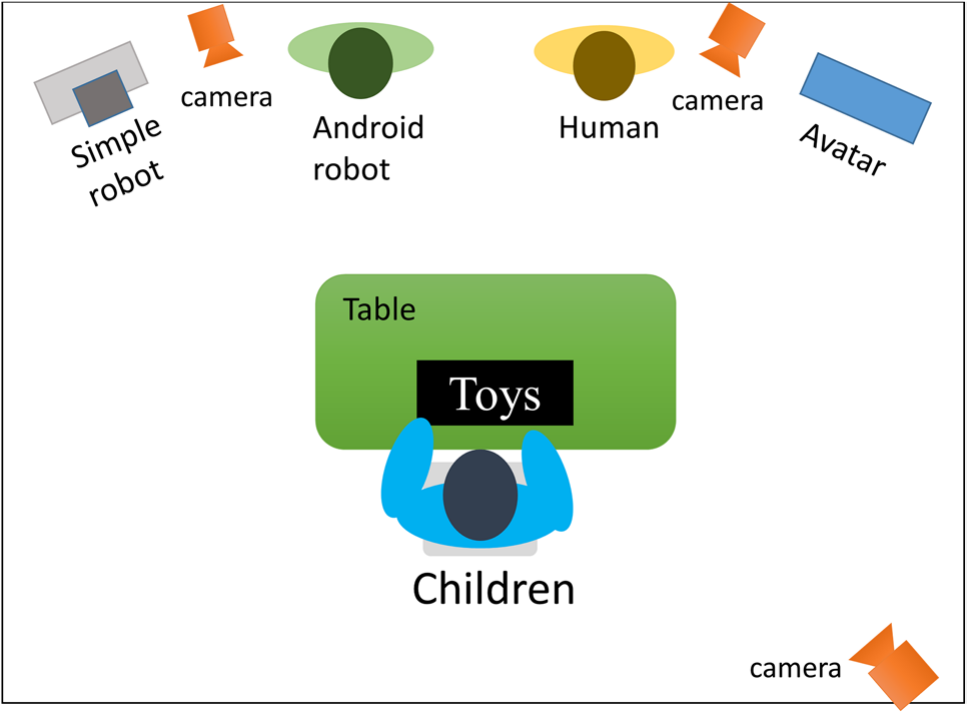
The experimental room setting

Participants were individually brought to an assessment room with their caregivers who remained with their child during the session. A child-sized table and chairs were set up in the middle of the assessment room with a variety of toys. Parents were invited to sit with their child and play with the available toys. Approximately 8 feet in front of the table where the child was seated, the three technological agents and human assistant were seated along the perimeter of the room. The technological agents were live before the child entered the room (i.e., two robots in the seated position with eyes open and face forward; avatar’s face visible on screen with eyes open) and the human assistant was seated quietly, face forward with a neutral expression. A digital camera was set up by each agent to capture any participant response to social prompts for off-line analysis. Figure 4 illustrates experiment room setup.

For most children, it only took 1–3 min to be seated at the table and engage with the toys after entering the room. Study personnel were observing various emotions expressed by children, such as discomfort or fear regarding the technical agents. During the process of gathering informed consents, parents were encouraged to let the study personnel know if they identified stress in their child. The administration of social presses began once the child was engaged in play after 1–3 min. The three technological agents and human assistant provided a verbal social bid of “Hey!” at each trial. Two humanoid robots provided a verbal social bid of “Hey!” from behind each agent. A screen-based digital avatar provided a verbal social bid of “Hey!” from the exact location where the face appeared, that is, a personal computer with a built-in speaker.

The first author sat at the table where the child was going to sit and measured the volume of each technological and human agent using a dB meter (SkyPaw Co. Ltd). If the volume of a technological agent was not 56 dB, the operator (i.e., another research assistant) changed its volume. If the volume of a human agent was not 56 dB, the first author instructed human agent to increase or decrease the volume. We repeated these trials several times until the volume of each agent was controlled to around 56 dB. The social bid was presented in two conditions: high and low. The low condition consisted only of the social phrase “Hey!” directed toward the child with a slight, naturalistic movement of the head and neck (e.g. a nod). The high condition consisted of the same social phrase “Hey!” directed toward the child, paired with a larger gesture. For the android robot and digital avatar, the larger gesture consisted of bending forward and inclining its head in the direction of the child (e.g., leaning closer with a more pronounced nod of the head). For the human assistant and simple humanoid robot, the larger gesture consisted of raising a hand and inclining the head in the direction of the child (e.g., waving with a nod of the head). Thirty-two trials were presented (eight trials from each presenter; four in the high-condition; four in the low-condition). Each trial was separated by approximately five seconds and presented in a random order. It was possible, depending on the randomized order generated for each session, for the same agent to repeat the social prompt two or more times in a row. We did not employ a rule requiring that all four agents take turns before resuming the next four trials. In our system, three technological agents were operated by the same research assistant. To match the timing of the verbal social bid by each technological agent with the scheduled timings, the assistant pressed a button while watching a stopwatch. For the human agent to do it, the assistant counted five seconds in her mind after the last agent called “Hey!” and provided a social bid of “Hey!” In a preliminary experiment, we practiced this repeatedly to ensure that the four agents provided a verbal social bid of “Hey” toward the participant every five seconds exactly.

To elicit the appearance that the robots were behaving and reacting autonomously, we adopted a remote control system similar to those conventionally used in robotics research (Nishio et al. 2013). Throughout the session the three technological agents were tele-operated from an adjacent room divided by a one-way mirror. Parents were invited to sit with their child throughout the session, and to respond naturally with a smile or nod if the child engaged the parent during the free play activity with available toys, but to strictly avoid directing the child’s attention to all the agents at the front of the room. Later video coding confirmed that all parents adhered to these guidelines. Being mindful that the robots could elicit a fear response in certain children, the parent and human assistant were asked to be observant of any anxiety or fear during the session so that trials could be discontinued if the child became afraid. The child’s response to each social bid was measured off-line by counting the frequency with which the child turned his or her head and/or eyes toward the agent following the social press. Two independent raters assessed the experimental videos. The primary rater was blind to the participant group and study objective. The secondary rater participated throughout the experiment and was familiar with the objective of this study and the group of the participants (ASD or TD). The primary and secondary raters attained a high degree of reliability [intra-class coefficient (ICC) = 0.98]. The score reported in the current manuscript was derived by the primary rater.

### Data Analysis

We performed statistical analysis using SPSS version 24.0 (IBM, Armonk, NY, USA). Descriptive statistics for the sample were used. First, in order to investigate the differences in the proportions of turning of the head to various agents between children with ASD and TD, we employed two-way ANOVA with “diagnosis; 2 levels (1, TD; 2, ASD)” as the between-group factor and “agent; 4 levels (1, Android robot; 2, Simple robot; 3, Avatar on the display; 4, Human)” as the within-group factor. Second, depending on the type of agent, to see whether the response of a child was different, we employed one-way ANOVA in which “agent; 4 levels (1, Android robot; 2, Simple robot; 3, Avatar on the display; 4, Human)” as within-group factor was used separately in children with TD and ASD. Third, as post hoc analysis, Fisher PLSD tests were performed to ascertain whether a particular agent yielded higher responses than another agent in children with ASD and/or TD. Pearson product-moment correlation coefficients were used to explore the relationships between age, SRS-2, SCQ, CDI, and response to each agent in children with ASD. An alpha level of 0.05 was employed for these analyses.

## Results

### Demographic Data

There were no significant differences between groups with regards to mean age (*t* = 0.31; *p* = 0.76; *d* = 0.05) and gender proportion (χ2 = 0.82; *p* = 0.37), by using an independent samples t-test and a χ2-test respectively. As expected, there were significant differences with regard to SRS-2 (*t* = 11.72; *p* < 0.01; *d* = 0.89) and SCQ (*t* = 10.63; *p* < 0.01; *d* = 0.87) and CDI (*t* = − 3.45; *p* < 0.01; *d* = 0.50) between groups of children with ASD and TD by using an independent samples t-test. Participant details are presented in Table 1.

### Percentage of Head Turns to Each Agent

Percentage of head turns of the ASD group were significantly lower than that of the TD group toward the android robot (24.1 ± 28.8 vs 77.2 ± 26.6; *t* = − 5.71; *p* < 0.001; *d* = 0.69), simple humanoid robot (40.2 ± 29.9 vs. 79.9 ± 22.8; *t* = − 4.56; *p* < 0.001; *d* = 0.61), avatar on the computer (35.7 ± 31.7 vs. 84.8 ± 19.2; *t* = − 5.83; *p* < 0.001; *d* = 0.70), and human (25.9 ± 24.7 vs. 88.6 ± 15.0; *t* = − 9.62; *p* < 0.001; *d* = 0.83), by using an independent samples t-test (Table 2). The two-way ANOVA resulted in significant interaction effect between the group and conditions (*F* = 4.897; *p* = 0.003; *η*^*2*^_*G*_ = 0.522). One-way ANOVA resulted in significant condition effect in children with ASD (*F* = 5.538; *p* = 0.029; *η*^*2*^_*G*_ = 0.043) and TD (*F* = 3.549; *p* = 0.019; *η*^*2*^_*G*_ = 0.055). Therefore, depending on the type of agent, the proportions of turning of the head are different in children with TD and ASD.

**Table 2:** Distribution of turn head per sound category

Group	ASD (n = 14) mean (SD)	TD (n = 23) mean (SD)	Statistics		
			*t*	*p*	*d*
Android robot	24.1 (28.8)	77.2 (26.6)	− 5.71	< 0.001	0.69
Simple robot	40.2 (29.9)	79.9 (22.8)	− 4.56	< 0.001	0.61
Avatar on the display	35.7 (31.7)	84.8 (19.2)	− 5.83	< 0.001	0.70
Human	25.9 (24.7)	88.6 (15.0)	− 9.62	< 0.001	0.83

Numbers indicate corresponding percentage of turn-head for auditory stimulation

*SD* Standard deviation, *ES* effect size

Post hoc analysis revealed that, in children with ASD, the proportion of turning of the head to the human was significantly lower than that toward the simple humanoid robot (*t* = − 2.66; *p* = 0.020; *d* = 0.72) or the avatar (*t* = − 2.15; *p* = 0.049; *d* = 0.51). The proportion of turning of the head to the simple humanoid robot was significantly higher than that in response to the android robot (*t* = 3.03; *p* = 0.010; *d* = 0.64). The proportion of turning of the head to the avatar was significantly higher than that in response to the android robot (*t* = 3.85; *p* = 0.004; *d* = 0.73). There were no significant differences in the turning of the head towards the android robot and the human (*t* = − 0.52; *p* = 0.612; *d* = 0.14), and the simple humanoid robot and the avatar (*t* = 0.84; *p* = 0.418; *d* = 0.23).

In children with TD, the proportion of turning of the head to the human was significantly higher than that towards the android robot (*t* = 2.61; *p* = 0.016; *d* = 0.49) or the simple humanoid robot (*t* = 2.29; *p* = 0.032; *d* = 0.44). There were no significant differences in the turning of the head towards the android robot and simple humanoid robot (*t* = − 0.72; *p* = 0.478; *d* = 0.15), android robot and the avatar (*t* = − 1.806; *p* = 0.085; ES = 0.36), simple humanoid robot and the avatar (*t* = − 1.44; *p* = 0.165; *d* = 0.29), and the avatar and the human (*t* = − 1.19; *p* = 0.245; *d* = 0.25).

We did not find any relationship between the responses to each agent and age (android robot: *r* = 0.31, *p* = 0.28, simple humanoid robot: *r* = 0.20, *p* = 0.50, avatar on the computer: *r* = 0.20, *p* = 0.49, human: r = − 0.03, *p* = 0.91), SRS-2 (android robot: *r* = − 0.07, *p* = 0.87, simple humanoid robot: *r* = 0.06, *p* = 0.88, avatar on the computer: *r* = − 0.14, *p* = 0.73, human: *r* = − 0.17, *p* = 0.67), SCQ (android robot: *r* = − 0.22, *p* = 0.52, simple humanoid robot: *r* = − 0.02, *p* = 0.96, avatar on the computer: *r* = − 0.32, *p* = 0.33, human: *r* = − 0.32, *p* = 0.34), and CDI (android robot: *r* = 0.50, *p* = 0.07, simple humanoid robot: *r* = 0.62, *p* = 0.15, avatar on the computer: *r* = 0.35, *p* = 0.23, human: *r* = 0.13, *p* = 0.65), in children with ASD.

## Discussion

In the current study, we examined differences between children with ASD and TD in their responses to social bids presented by both human assistant and a set of distinct technological agents. These technological agents ranged from a robot bearing remarkably close similarity to humans, to one clearly non-human/mechanical, as well as a screen-based model—a digital avatar common to virtual reality systems used in both gaming and intervention. While simple in its design, the aim was to provide preliminary data regarding which agent was best at eliciting a response to a social cue from children with and without ASD in hopes of designing appropriate and tailored robotic assessment/intervention paradigms over time.

As hypothesized, clear differences were observed between children with ASD and TD in their response to social calls, which is in line with the findings of previous studies (Dawson et al. 2004; Swettenham et al. 1998). Independent of the agent, children with ASD responded notably less to social bids coming collectively from the two robots, avatar and human compared to responses by their typically developing peers. In addition, some differences of interest emerged when we compared response rates of individual groups (ASD vs. TD) to the four agents. Children with ASD showed a significant preference to the simple humanoid robot and the screen-based avatar, and responded less to the social bids coming from the human and “human-like” android robot. As discussed, previous research has suggested that children with ASD may demonstrate attentional differences and/or prefer certain types of interactions with robots when compared to human partners (Pierno et al. 2008) and show enhanced performance in response to robotic presses compared to presses delivered by a human confederate (Warren et al. 2015). The current study supports this trend, but also highlights the reality that not all types of technological and robotic interactions will demonstrate such preference. In fact, within the current work when robotic systems approached human qualities of appearance and function, attentional differences and preferential response patterns were not significant. We originally expected that an android system would also document differences in response. However, the lower impact of the android’s success rate was in line with some qualitative reports of an unease invoked by adults in the presence of an android (Glass 2017). In fact, preschoolers also responded the least amount to android robot, further indicating that this type of robot could be less suited for successful incorporation into robot-assisted intervention activities with young children. The result could be explained by the concept of the uncanny valley (Mori 1970) which holds that a human-looking robot can provoke repulsion and sensations of eeriness. However, other explanations warrant consideration and discussion. There is the possibility that the android was simply perceived as another person, and just as the children with ASD did not attend to the human assistant, they may similarly have avoided their gaze toward the android. Future work examining the child’s interpretation of the android (android robot vs. human) would help clarify similarities in the reactions towards the android and human. The typically developing children in our study responded the most to social presses given by the human and a significant difference was not observed between responses to the digital avatar versus human. These results may be understood considering that children are accustomed to looking at characters on screens. It is likely common for TD preschool children to frequently respond to a voice on a television monitor compared to interacting/responding to a physical robot bidding for their attention.

A potential benefit of this study is that the age range of participants was markedly younger (average age of 3 years) compared to that of previous work utilizing robot-assisted tasks or intervention. As toddlers and preschool children represent a common age for intervention tasks to best focus on key social deficits, it was useful to explore initial response rates from young children when exposed to various technical agents.

While the current study was purposefully simple in design, several limitations are worth consideration. Children were only seen for one session and the session time was short in duration in order to capture their preferences over a predetermined number of verbal prompts. Sessions over a longer time span may offer more extensive understanding of habituation to each technical agent over time, as well as allow for a variety of social prompts or interactions to be used and observed beyond a friendly greeting (i.e. “Hey!”). While the current study leaves us with data suggesting that the simple humanoid robot was best at provoking a look from the children with ASD, we are unable to comment on whether this would hold for higher demands such as imitation tasks, conversation, or emotion recognition which would lead to meaningful change across environments. This said, as a pilot study we were pleased in the strong success rate that the children with ASD demonstrated in terms of remaining in the assessment area and engaging in the activity as designed (i.e. playing with available materials and responding to agents’ calls when desired). All participants except for one child with ASD fully completed the experimental session. Future comparative studies could certainly build on the present work in order to broaden our understanding of the utility of robot types in intervention activities. Second, our study employed a relatively small number of participants (n = 38), which included 23 TD children and 15 children with ASD. Larger sample sizes, particularly in the ASD group, would be beneficial. Third, the method used to obtain an objective measure of relative preference for social sounds was the observation of eye-gaze or head-turn toward the person/agent giving the social bid. Though this “head-turn method” has been the most common means to investigate the attention to sound (Gilbertson et al. 2016), it relies heavily on the assumption of a strong association between preference and orientation to a sound, when in fact the child’s attention to the social sound may be provoked for a variety of reasons unrelated to preference (e.g., distraction, surprise, or even annoyance) (Gilbertson et al. 2016). Again, observation over multiple sessions with a variety of social presses may help further support sustained preference versus short-range attention. Yet attentional preferences for robotic interactions hold significance when considering intervention strategies to promote change in core skills tied to core communicative and social deficits associated with autism spectrum disorders (Robins et al. 2005). Fourth, the locations of the agents were not counterbalanced. This was due to the technical restraints within the experimental room regarding positioning of the android robot and avatar. We attempted to help mediate the lack of counterbalancing by positioning the children and toys at a distance far enough from the agents so that eye gaze could occur naturally, oriented to the front of the room, and regardless of the position. That is, all four agents were in the central range of focus if the child looked up in response to a social bid, rather than in the periphery, which would require a clear head shift to the right or left. In this study, the results indicated that simple humanoid robot and the avatar (placed in positions 1 and 4, respectively) elicited a higher rate of response in children with ASD.

In sum, considering an established attentional preferences to robots among children with ASD (Bekele et al. 2013; Duquette et al. 2007; Kumazaki et al. 2017c; Robins et al. 2009), the current work offers a novel comparison between different robot types and technological agents, suggesting that type of technological system likely matters a great deal for different children and approaches. Full utilization of robotic technologies in intervention settings will require specific attention to these differences in order to better understand the suitability of specific robot types for therapeutic use. Further work investigating the real impact on specific social and communication deficits with the help of robot-assisted therapies within a controlled clinical approach may prove to be both beneficial and well-timed.

## References

[CR1] American Psychiatric Association (2013). Diagnostic and statistical manual of mental disorders.

[CR2] Annaz D, Campbell R, Coleman M, Milne E, Swettenham J (2011). Young children with autism spectrum disorder do not preferentially attend to biological motion. Journal of Autism and Developmental Disorders.

[CR3] Anzalone SM (2014). How children with autism spectrum disorder behave and explore the 4-dimensional (spatial 3D + time) environment during a joint attention induction task with a robot. Research in Autism Spectrum Disorders.

[CR4] Bekele E, Crittendon JA, Swanson A, Sarkar N, Warren ZE (2013). Pilot clinical application of an adaptive robotic system for young children with autism. Autism.

[CR5] Bernard-Opitz V, Sriram N, Nakhoda-Sapuan S (2001). Enhancing social problem solving in children with autism and normal children through computer-assisted instruction. Journal of Autism and Developmental Disorders.

[CR6] Constantino J, Gruber C (2002). The Social Responsiveness Scale.

[CR7] Cook J, Swapp D, Pan X, Bianchi-Berthouze N, Blakemore SJ (2014). Atypical interference effect of action observation in autism spectrum conditions. Psychological Medicine.

[CR8] Costa S, Lehmann H, Dautenhahn K, Robins B, Soares F (2014). Using a humanoid robot to elicit body awareness and appropriate physical interaction in children with autism. International Journal of Social Robotics.

[CR9] Dautenhahn K (2007). Socially intelligent robots: Dimensions of human-robot interaction. Philosophical Transactions of the Royal Society B: Biological Sciences.

[CR10] Dawson G (2004). Early social attention impairments in autism: Social orienting, joint attention, and attention to distress. Developmental Psychology.

[CR11] Dawson G (2008). Early behavioral intervention, brain plasticity, and the prevention of autism spectrum disorder. Development and Psychopathology.

[CR12] Diehl JJ, Schmitt LM, Villano M, Crowell CR (2012). The clinical use of robots for individuals with autism spectrum disorders: A critical review. Research in Autism Spectrum Disorders.

[CR13] Duquette A, Michaud F, Mercier H (2007). Exploring the use of a mobile robot as an imitation agent with children with low-functioning autism. Autonomous Robots.

[CR14] Durkin K (2010). Videogames and young people with developmental disorders. Review of General Psychology.

[CR15] Fenson L, Marchman V,A, Thal D,J, Philip S, Steven. DJ, Elizabeth R (2007). MacArthur-Bates communicative development inventories: User’s guide and technical manual.

[CR16] Gilbertson LR, Lutfi RA, Ellis Weismer S (2016). Auditory preference of children with autism spectrum disorders. Cognitive Processing.

[CR17] Glass, N. (2017). What the faces of our robots tell us about ourselves. CNN definitive design, 12 May 2017. Retrieved September 10, 2017, from http://www.cnn.com/style/article/science-museum-robots-design/index.html.

[CR18] Goodrich, M. A., Colton, M. A., Brinton, B., & Fujiki, M. (2011). A case for low-dose robotics in autism therapy. In *Proceedings of the 6th international conference on human-robot interaction* (p. 143). 10.1145/1957656.1957702.

[CR19] Goodwin MS (2008). Enhancing and accelerating the pace of autism research and treatment. Focus on Autism and Other Developmental Disabilities.

[CR20] Grynszpan O, Weiss PL, Perez-Diaz F, Gal E (2013). Innovative technology-based interventions for autism spectrum disorders: A meta-analysis. Autism.

[CR21] Iacono, I., Lehmann, H., Marti, P., Robins, B., & Dautenhahn, K. (2011). Robots as social mediators for children with Autism—A preliminary analysis comparing two different robotic platforms. In *2011 IEEE international conference on development and learning (ICDL)* (pp. 1–6). 10.1109/devlrn.2011.6037322.

[CR22] Kim ES (2012). Social robots as embedded reinforcers of social behavior in children with autism. Journal of Autism and Developmental Disorders.

[CR23] Klin A, Lin DJ, Gorrindo P, Ramsay G, Jones W (2009). Two-year-olds with autism orient to non-social contingencies rather than biological motion. Nature.

[CR24] Kumazaki H (2017). Tele-operating an android robot to promote the understanding of facial expressions and to increase facial expressivity in individuals with autism spectrum disorder. American Journal of Psychiatry.

[CR25] Kumazaki H (2017). Android robot-mediated mock job interview sessions for young adults with autism spectrum disorder: A pilot study. Frontiers in Psychiatry.

[CR26] Kumazaki H (2017). A pilot study for robot appearance preferences among high-functioning individuals with autism spectrum disorder: Implications for therapeutic use. PLoS ONE.

[CR27] Kumazaki H (2017). Impressions of humanness for android robot may represent an endophenotype for autism spectrum disorders. Journal of Autism and Developmental Disorders.

[CR28] Lee, J., & Obinata, G. (2013). Developing therapeutic robot for children with autism: A study on exploring colour feedback. In *Proceedings of the 8th ACM/IEEE international conference on human-robot interaction* (pp. 173–174). 10.1109/hri.2013.6483557.

[CR29] Lee, J., Takehashi, H., Nagai, C., & Obinata, G. (2012a). Design of a therapeutic robot for interacting with autistic children through interpersonal touch. 10.1109/roman.2012.6343835.

[CR30] Lee J, Takehashi H, Nagai C, Obinata G, Stefanov D (2012). Which robot features can stimulate better responses from children with autism in robot-assisted therapy?. International Journal of Advanced Robotic Systems.

[CR31] Lord C, Rutter M, DiLavore PC, Risi S, Gotham K, Bishop S (2012). Autism diagnostic observation schedule, second edition (ADOS-2).

[CR32] Moore M, Calvert S (2000). Brief report: Vocabulary acquisition for children with autism: Teacher or computer instruction. Journal of Autism and Developmental Disorders.

[CR33] Mori M (1970). Bukimi no tani [The uncanny valley]. Energy..

[CR34] Mundy P, Gwaltney M, Henderson H (2010). Self-referenced processing, neurodevelopment and joint attention in autism. Autism.

[CR35] Nishio S, Taura K, Sumioka H, Ishiguro H (2013). Teleoperated android robot as emotion regulation media. International Journal of Social Robotics.

[CR36] Peca A, Simut R, Pintea S, Costescu C, Vanderborght B (2014). How do typically developing children and children with autism perceive different social robots?. Computers in Human Behavior.

[CR37] Pennisi P (2016). Autism and social robotics: A systematic review. Autism Research.

[CR38] Pierno AC, Mari M, Lusher D, Castiello U (2008). Robotic movement elicits visuomotor priming in children with autism. Neuropsychologia.

[CR39] Ploog BO, Scharf A, Nelson D, Brooks PJ (2012). Use of computer-assisted technologies (CAT) to enhance social, communicative, and language development in children with autism spectrum disorders. Journal of Autism and Developmental Disorders.

[CR40] Poon KK, Watson LR, Baranek GT, Poe MD (2011). To what extent do joint attention, imitation, and object play behaviors in infancy predict later communication and intellectual functioning in ASD?. Journal of Autism and Developmental Disorders.

[CR41] Ricks, D. J., & Colton, M. B. (2010). Trends and considerations in robot-assisted autism therapy. In *2010 IEEE international conference on robotics and automation (ICRA)* (pp. 4354–4359). 10.1109/robot.2010.5509327.

[CR42] Robins B, Dautenhahn K, Boekhorst RT, Billard A (2005). Robotic assistants in therapy and education of children with autism: Can a small humanoid robot help encourage social interaction skills?. Universal Access in the Information Society.

[CR43] Robins, B., Dautenhahn, K., & Dickerson, P. (2009). From isolation to communication: A case study evaluation of robot assisted play for children with autism with a minimally expressive humanoid robot. In *Second international conferences on advances in computer-human interactions, 2009. ACHI’09* (pp. 205–211). 10.1109/achi.2009.32.

[CR44] Rutter M, Bailey A, Lord C (2010). The social communication questionnaire.

[CR45] Shimaya, J., et al. (2016). Advantages of indirect conversation via a desktop humanoid robot: Case study on daily life guidance for adolescents with autism spectrum disorders. In *2016 25th IEEE international symposium on robot and human interactive communication (RO-MAN)* (pp. 831–836) 10.1109/roman.2016.7745215.

[CR46] Swettenham J (1998). The frequency and distribution of spontaneous attention shifts between social and nonsocial stimuli in autistic, typically developing, and nonautistic developmentally delayed infants. Journal of Child Psychology and Psychiatry, and Allied Disciplines.

[CR47] Wainer J, Dautenhahn K, Robins B, Amirabdollahian F (2013). A pilot study with a novel setup for collaborative play of the humanoid robot KASPAR with children with autism. International Journal of Social Robotics.

[CR48] Wainer J, Ferrari E, Dautenhahn K, Robins B (2010). The effectiveness of using a robotics class to foster collaboration among groups of children with autism in an exploratory study. Personal and Ubiquitous Computing.

[CR49] Wainer J, Robins B, Amirabdollahian F, Dautenhahn K (2014). Using the humanoid robot KASPAR to autonomously play triadic games and facilitate collaborative play among children with autism. IEEE Transactions on Autonomous Mental Development.

[CR50] Warren Z (2015). Brief report: Development of a robotic intervention platform for young children with ASD. Journal of Autism and Developmental Disorders.

[CR51] Yin, T.-C., & Tung, F.-W. (2013). Design and evaluation of applying robots to assisting and inducing children with autism in social interaction. *International conference on universal access in human-computer interaction* (Vol. 8010, pp. 524–533). 10.1007/978-3-642-39191-0_57.

